# Reclassifying contralateral axillary metastasis: survival benefit of aggressive local therapy challenges stage IV designation

**DOI:** 10.3389/fonc.2026.1748851

**Published:** 2026-04-16

**Authors:** Shanqing Liu, Yong Li, Yan Shen, Qixin Mao, Lianfang Li

**Affiliations:** Department of Breast Disease, Henan Breast Cancer Centre, The Affiliated Cancer Hospital of Zhengzhou University and Henan Cancer Hospital, Zhengzhou, China

**Keywords:** breast cancer, Contralateral axillary metastasis, Locoregional therapy, overall survival, staging

## Abstract

**Background:**

Contralateral axillary metastasis (CAM) is currently staged as M1 (stage IV) breast cancer, guiding treatment towards palliative systemic therapy. However, emerging evidence suggests CAM may behave as a locoregional event, potentially amenable to curative-intent strategies. This study aims to define the prognosis of isolated CAM and evaluate the survival impact of aggressive local therapy.

**Methods:**

We conducted a retrospective cohort study of 1110 patients from two tertiary centers, comprising three cohorts: CAM (n=128), stage IIIC locally advanced breast cancer (LABC, n=532), and *de novo* oligometastatic (M1, n=450) disease. Overall survival (OS) was compared using Kaplan-Meier and multivariable Cox regression. Within the CAM cohort, we employed propensity score matching (PSM), inverse probability of treatment weighting (IPTW), and multivariable Cox models to compare outcomes between patients receiving curative-intent local therapy (surgery/radiotherapy) versus a systemic therapy-focused approach.

**Results:**

The adjusted mortality risk for the CAM cohort was significantly lower than for the M1 cohort (aHR 0.58, 95% CI 0.45–0.74) but higher than for the LABC cohort (aHR 1.18, 95% CI 0.93–1.60). In the PSM analysis, curative-intent therapy was associated with a 51% reduction in mortality risk (HR 0.49, 95% CI 0.31–0.78) and a significantly lower 5-year cumulative incidence of locoregional recurrence (12.1% vs. 31.4%, p=0.005). Results were consistent across IPTW and multivariable analyses.

**Conclusion:**

Patients with isolated CAM exhibit a prognosis distinct from classic stage IV disease. A curative-intent local treatment strategy is independently associated with significantly improved survival and locoregional control, supporting the re-evaluation of CAM as a clinically regionally treatable entity.

## Introduction

Contralateral axillary metastasis (CAM), defined as the presence of metastatic breast cancer in the lymph nodes of the axilla opposite to the primary breast tumor, presents a significant therapeutic dilemma in clinical oncology (1). Its classification and optimal management remain subjects of intense debate. The American Joint Committee on Cancer (AJCC) staging system classifies CAM as stage IV (M1) disease, categorizing it as a distant metastatic event. This classification inherently guides treatment towards a palliative, systemic therapy-focused paradigm, similar to that used for visceral metastases (2). However, a growing body of evidence challenges this dogma, suggesting that CAM may represent a manifestation of progressive locoregional disease rather than systemic dissemination, particularly when it occurs in isolation without other distant metastases (3).

The rationale for re-evaluating the classification of CAM stems from emerging clinical data demonstrating that patients with isolated CAM who are treated with aggressive, multimodal therapy with curative intent exhibit survival outcomes that diverge markedly from those of patients with classic stage IV disease (4). The seminal study by Nash et al. (5), utilizing the NCDB provided large-scale evidence for this paradigm shift. Their analysis showed that patients with CAM treated with surgery and radiation had overall survival (OS) comparable to patients with locally advanced breast cancer (LABC) and significantly superior to those with M1 disease. These findings led the authors to propose that CAM should be reclassified as N3 disease, reflecting a regional rather than a distant process. This conclusion was recently bolstered by a contemporary multi-institutional analysis by Flanagan et al. (6), which further confirmed that patients with CAM treated with curative intent had OS similar to an LABC cohort and significantly improved compared to a metastatic breast cancer (MBC) cohort.

Conversely, not all evidence uniformly supports this view. A study by Zhao et al. (7) proposed an alternative perspective, positioning CAM as an “oligometastatic-like” disease. Their single-institution analysis found that the survival of patients with CAM was comparable to those with oligometastasis (OM) and inferior to those with locoregional recurrence (LRR), leading them to suggest that systemic therapy, not aggressive local treatment, should be the cornerstone of management. This conflicting evidence highlights a critical knowledge gap and underscores the lack of consensus on whether the potential survival benefit associated with aggressive local therapy for CAM is a result of the treatment itself or of selection bias, where patients with more favorable tumor biology are chosen for such intensive approaches (8).

Therefore, the central question persists: Does CAM represent a regional event amenable to curative-intent strategies, or is it a systemic disease where local therapy offers little benefit? To address this, we conducted a retrospective cohort study harmonizing data from two tertiary academic centers. Our study aims to robustly situate the prognosis of CAM within the breast cancer spectrum by directly comparing outcomes of a well-defined CAM cohort with two carefully selected comparator cohorts: a cohort with Stage IIIC LABC and a cohort with *de novo* oligometastatic (M1) disease. Furthermore, we employed propensity score methodologies to minimize confounding and explicitly evaluate the survival impact of a curative-intent local treatment strategy versus a systemic therapy-focused approach within the CAM cohort. By providing this comparative analysis and rigorously assessing the treatment effect, our study seeks to deliver high-quality evidence to inform clinical practice and potentially guide future revisions to staging systems.

## Methods

### Study design

This was a retrospective cohort study conducted using harmonized electronic health record data from two tertiary academic cancer centers (The Affiliated Cancer Hospital of Zhengzhou University and The First Affiliated Hospital of Zhengzhou University). The study period spanned from January 1, 2005, to December 31, 2022, a design which allowed for a minimum potential follow-up of three years for all included subjects. The study received approval from the Institutional Review Board at each participating site, which granted a waiver of informed consent for the use of retrospective, de-identified data.

### Patient population

The study population consisted of three distinct cohorts derived from the same institutional databases. First, the CAM Cohort included adult patients (aged 18 years or older) with cytologically or histologically confirmed CAM. This cohort was further stratified into two subgroups based on the timing of CAM presentation: the Synchronous CAM subgroup, comprising patients in whom CAM was identified at the time of initial breast cancer diagnosis or before any definitive local therapy was initiated for the primary tumor; and the Metachronous CAM subgroup, consisting of patients for whom CAM was detected as the first site of disease recurrence after they had completed primary curative-intent treatment for early-stage breast cancer. To ensure a well-defined study sample, several exclusion criteria were applied to the CAM cohort. Patients were excluded if they had any radiological evidence of distant metastasis—such as involvement of bone, viscera, or non-axillary distant lymph nodes—at the time of CAM diagnosis, as determined by standard imaging modalities. Additionally, individuals with incomplete medical records pertaining to staging, treatment details, or follow-up status were excluded, as were those with a history of any other invasive malignancy within the 5 years preceding the CAM diagnosis, with the exception of non-melanoma skin cancer.

To better contextualize the prognostic implications of a CAM diagnosis, two additional comparator cohorts were established. The Locally Advanced Breast Cancer (LABC) Comparator Cohort consisted of patients diagnosed with Stage IIIC (cN2-N3, M0) breast cancer, staged according to the AJCC 8th edition as appropriate for the year of diagnosis. Patients in this cohort were required to have received comprehensive multimodal therapy with curative intent, mirroring the treatment approach of the Curative-Intent CAM group; this included definitive breast surgery, radiotherapy, and systemic therapy. A second comparator cohort was composed of patients with *de novo* Stage IV (M1) breast cancer who presented with a single, solitary metastatic lesion in the absence of other metastatic sites. This specific definition of a “single metastasis” was chosen for two key methodological reasons. First, it establishes a comparator group with a similarly limited disease burden to the “isolated” CAM cohort, allowing for a more equitable comparison of outcomes. Second, by restricting the cohort to *de novo* M1 patients, we avoid lead-time bias and the profound prognostic heterogeneity associated with disease-free interval that would be introduced by including patients with recurrent metastatic disease. Patients in this cohort were treated primarily with systemic therapy. The establishment of these two comparator cohorts allows for a robust analysis of whether the survival outcomes of patients with CAM align more closely with those of locally advanced disease or with distant metastatic disease.

### Variable definition

A standardized data abstraction form was implemented across all participating sites to ensure the consistent collection of comprehensive clinical data from the electronic health records. The collected variables were broadly categorized to capture a complete clinical picture of each patient’s journey. This included detailed patient demographics such as age at CAM diagnosis, sex, self-reported race and ethnicity, and the Charlson Comorbidity Index score to quantify the burden of concomitant illness. Furthermore, primary tumor characteristics were meticulously recorded, encompassing histologic type, grade, and the vital biomarker statuses for estrogen receptor (ER), progesterone receptor (PR), and HER2, the latter interpreted according to the ASCO/CAP guidelines contemporaneous to the original diagnosis, along with the Ki-67 index when available. The disease stage at initial presentation was documented using the Clinical and Pathological TNM classifications from the AJCC 7th or 8th edition, as was appropriate for the year of diagnosis.

Specific characteristics of the contralateral axillary metastasis were a central focus of the data collection. For each patient, the cohort (Synchronous or Metachronous) was defined. For those in the Metachronous CAM cohort, the Disease-Free Interval (DFI) was calculated as the time from the completion of primary curative-intent treatment to the date of CAM diagnosis. The method of CAM diagnosis—whether by imaging-guided fine-needle aspiration, core biopsy, or surgical dissection—and the number of pathologically involved contralateral axillary nodes were also abstracted. Treatment data were collected with particular attention to the therapeutic approach following CAM diagnosis. This included detailed information on all systemic therapy, noting the type, timing relative to the primary cancer, and the line of therapy relative to the CAM. Critically, patients were classified into one of two mutually exclusive groups based on the local therapy received for the CAM: the “Curative-Intent Group,” which comprised patients receiving multimodal therapy including definitive local treatment (surgical resection of the CAM and/or radiotherapy to the contralateral axilla with a curative dose ≥45 Gy), and the “Systemic Therapy-Focused Group,” for whom systemic therapy was the primary modality, with no specific or only palliative local therapy directed at the CAM.

Finally, comprehensive outcome data were collected to facilitate survival and recurrence analyses. This involved documenting the patient’s vital status and the date of last known follow-up. For deceased patients, the date and cause of death were ascertained to determine breast cancer-specific survival. Additionally, the date and anatomical site of the first documented recurrence or disease progression following the CAM diagnosis were recorded, with sites classified as either locoregional or distant. This rigorous and multi-faceted data collection strategy was designed to enable a robust comparison of treatment strategies and their associated long-term outcomes.

### Statistic analysis

Descriptive statistics were employed to summarize baseline patient, tumor, and treatment characteristics. Categorical variables are presented as frequencies and percentages, while continuous variables are summarized as medians with interquartile ranges (IQR). To compare characteristics across the three primary cohorts—CAM, LABC, and M1—the Kruskal-Wallis test was used for continuous variables and Chi-square or Fisher’s exact tests were applied for categorical variables. Additionally, standardized mean differences (SMD) were calculated to quantify the magnitude of baseline imbalance across cohorts, with an SMD of less than 0.1 indicating acceptable balance.

The primary analysis aimed to situate the prognosis of the entire CAM cohort within the spectrum of breast cancer stages. OS was defined as the time from the index date—which was the date of CAM diagnosis for the CAM cohort, and the date of initial diagnosis for both the LABC and M1 cohorts—to death from any cause. In the unadjusted analysis, Kaplan-Meier curves were generated to visualize OS for the three cohorts, and the log-rank test was used to compare their survival distributions. For the adjusted analysis, a multivariable Cox proportional hazards model was fitted to account for fundamental prognostic differences between these inherently distinct cohorts. This model included cohort membership (LABC versus CAM versus M1) as the primary variable of interest, while adjusting for key prognostic factors measured at the index date, including age, tumor grade, ER status, PR status, and HER2 status. The proportional hazards assumption was verified using Schoenfeld residuals.

A secondary analysis evaluated the association between local treatment strategy and survival specifically within the CAM cohort, where clinical equipoise exists. The association between treatment strategy (Curative-Intent versus Systemic Therapy-Focused) and OS was first assessed using unadjusted Kaplan-Meier curves and the log-rank test. To control for confounding and selection bias, a propensity score (PS) analysis was then conducted. This involved first fitting a multivariable logistic regression model to estimate each patient’s probability of receiving curative-intent therapy, incorporating clinically relevant covariates such as age, CAM cohort (synchronous or metachronous), disease-free interval (DFI), tumor subtype, and comorbidity score. Subsequently, propensity score matching (PSM) was performed using a 1:1 nearest-neighbor matching algorithm with a caliper width set at 0.2 of the standard deviation of the PS logit to create a balanced cohort, with balance assessed via SMD. As a sensitivity analysis, a pseudo-population was created using inverse probability of treatment weighting (IPTW) with stabilized weights. Kaplan-Meier analysis and Cox regression were then repeated in both the PS-matched and IPTW-weighted cohorts to estimate the treatment effect.

An exploratory analysis was performed to test the hypothesis that CAM behaves as a regional event. This involved comparing the survival outcomes of the Curative-Intent CAM Group with those of the LABC Comparator Cohort, and separately comparing the Systemic Therapy-Focused CAM Group with the Oligometastatic M1 Comparator Cohort. These comparisons were conducted using Kaplan-Meier analysis and multivariable Cox models adjusted for age and tumor subtype.

Patterns of treatment failure were analyzed by estimating the cumulative incidence of locoregional recurrence and distant metastasis. This was accomplished using the Cumulative Incidence Function (CIF), which appropriately treats death without the event of interest as a competing risk. Differences in cumulative incidence curves between the Curative-Intent and Systemic Therapy-Focused CAM groups were compared using Gray’s test. Furthermore, a multivariable Fine-Gray subdistribution hazards model was employed to identify factors independently associated with the risk of locoregional recurrence.

For the handling of missing data, the proportion of missingness was reported for all variables. For any variables included in the primary multivariable models that had more than 5% missing data, multiple imputation by chained equations was used to create 20 complete datasets. All statistical analyses were performed on each imputed dataset, and the results were subsequently pooled using Rubin’s rules. All analyses were conducted using R software (version 4.2.2), and a two-sided p-value of less than 0.05 was considered statistically significant.

## Results

### Baseline data

In total, 1110 patients were enrolled. The CAM cohort (n=128) was intermediate in age (median 55 years) between the younger LABC cohort (n=532, median 50 years) and the older M1 cohort (n=450, median 62 years, p<0.001). Tumor biology varied substantially across groups, with the CAM cohort exhibiting the highest proportion of grade 3 tumors (64.8%) compared to LABC (50.8%) and M1 (48.9%, p<0.001), while molecular subtype distribution showed significant but less pronounced variation (p=0.035). As expected by cohort definitions, dramatic differences emerged in initial nodal staging, with the LABC cohort comprising 100% cN2/N3 disease compared to 44.5% in the CAM cohort and 38.9% in the M1 cohort (p<0.001). Treatment patterns revealed fundamentally different approaches: both CAM and LABC cohorts received aggressive multimodal therapy with high rates of chemotherapy (93.8% and 96.1%), mastectomy (83.6% and 80.1%), and universal radiotherapy, whereas the M1 cohort was managed primarily with systemic therapy (70.0% chemotherapy) with minimal local intervention (22.0% mastectomy, 30.0% radiotherapy, p<0.001). The standardized mean differences further quantified these substantial imbalances, particularly highlighting the dramatic divergence in local treatment strategies between the CAM/LABC groups and the M1 cohort. (**Table 1**).

**Table 1:** Baseline characteristics of the propensity-score matched CAM cohort (n=86).

Characteristic	Curative-intent group (n=43)	Systemic therapy-focused group (n=43)	P-value	SMD
Molecular Subtype, n (%)			0.842	0.08
HR+/HER2-	21 (48.8%)	19 (44.2%)		
HER2+	11 (25.6%)	13 (30.2%)		
Triple-Negative	8 (18.6%)	7 (16.3%)		
Other/Unknown	3 (7.0%)	4 (9.3%)		
Age, median (IQR)	56 (48, 64)	55 (49, 63)	0.912	0.02
Synchronous CAM, n (%)	16 (37.2%)	15 (34.9%)	1.000	0.05
DFI (mo), median (IQR)	40 (26, 65)	43 (30, 70)	0.567	0.10
Charlson Index ≥2, n (%)	5 (11.6%)	6 (14.0%)	1.000	0.07

PSM, propensity score matching; SMD, standardized mean difference; HR, hormone receptor; CAM, contralateral axillary metastasis; DFI, disease-free interval. This table confirms excellent balance in molecular subtypes and other key covariates after matching.

### OS

**Figure 1:**
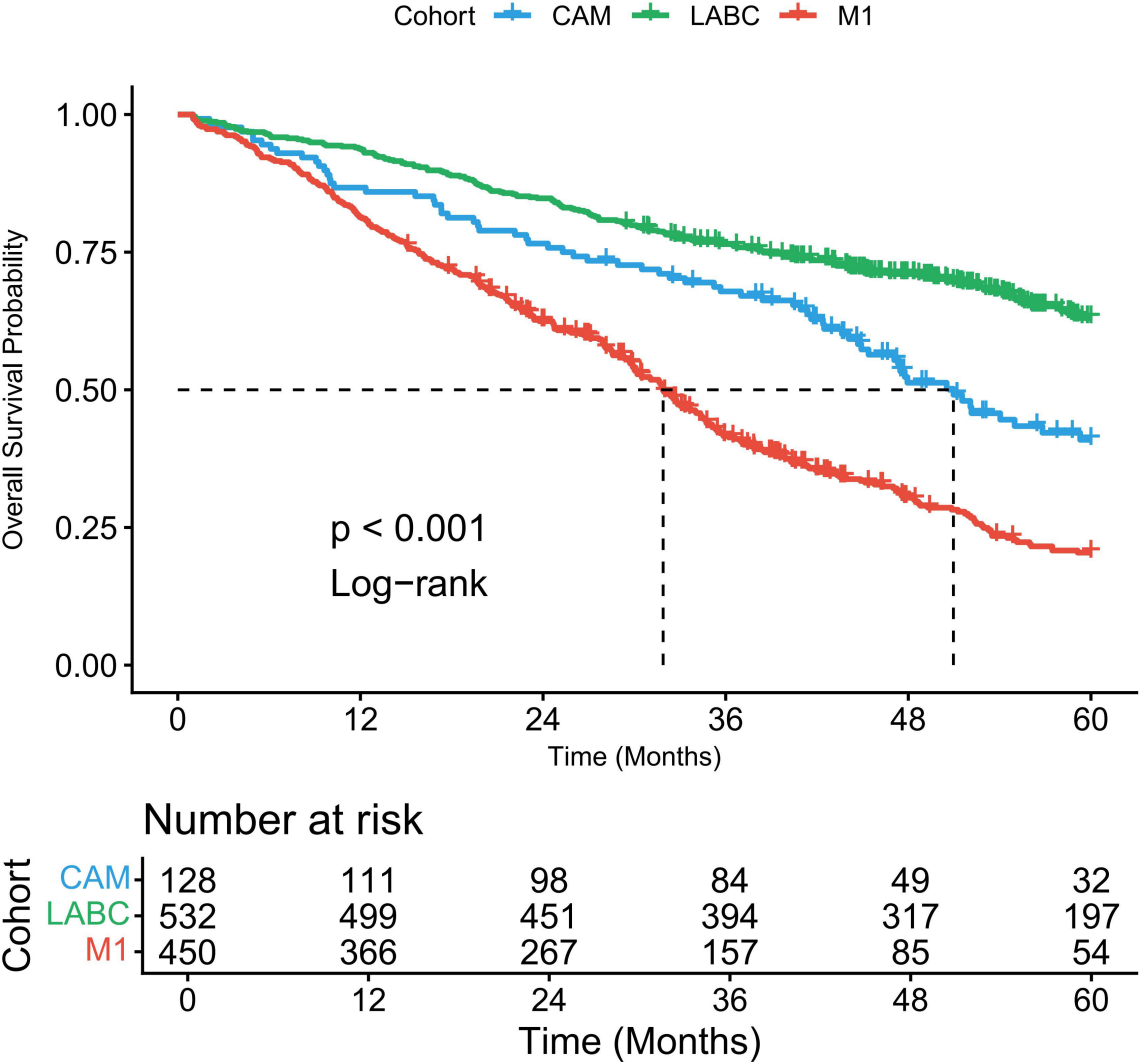
Overall survival comparison among CAM, LABC, and M1 cohorts. Kaplan-Meier curves showing overall survival for patients with contralateral axillary metastasis (CAM, n=128), stage IIIC locally advanced breast cancer (LABC, n=532), and *de novo* oligometastatic M1 breast cancer (n=450). Median overall survival was 48 months (95% CI, 41–56) for the CAM cohort, 72 months (95% CI, 65–80) for the LABC cohort, and 32 months (95% CI, 28–37) for the M1 cohort. Five-year overall survival rates were 44.2% (95% CI, 35.8–52.6%) for CAM, 58.7% (95% CI, 54.2–63.2%) for LABC, and 28.9% (95% CI, 24.3–33.5%) for M1. The CAM cohort demonstrated significantly better survival than the M1 cohort (log-rank p<0.001) and showed a trend toward inferior survival compared to the LABC cohort that did not reach statistical significance (adjusted HR 1.18, 95% CI 0.93–1.60; p=0.152). Tick marks indicate censored patients.

During the study period, a total of 776 deaths were observed: 89 deaths (69.5%) in the CAM cohort, 324 deaths (60.9%) in the LABC cohort, and 363 deaths (80.7%) in the M1 cohort. Median OS was 48 months (95% CI, 41–56) for the CAM cohort, 72 months (95% CI, 65–80) for the LABC cohort, and 32 months (95% CI, 28–37) for the M1 cohort. Five-year OS rates were 44.2% (95% CI, 35.8–52.6%), 58.7% (95% CI, 54.2–63.2%), and 28.9% (95% CI, 24.3–33.5%), respectively. The difference was significant (p<0.0001, **Figure 1**). In the unadjusted analysis, cohort membership was a powerful predictor of survival, with the CAM cohort demonstrating a significantly lower risk of death compared to the M1 cohort (HR 0.52, 95% CI 0.41–0.66, p<0.001) and the LABC cohort showing the most favorable prognosis (HR 0.43, 95% CI 0.36–0.51, p<0.001). After adjusting for key prognostic variables including age, comorbidity, and tumor characteristics, the association remained robust. After adjustment, the CAM cohort has a significantly lower risk of death than the M1 cohort (aHR 0.58, 95% CI 0.45--0.74). When directly compared to the LABC cohort, the CAM cohort shows a trend towards a higher risk of death (aHR 1.18, 95% CI 0.93--1.60), though this was not statistically significant. Other factors independently associated with worse overall survival in the multivariable model included older age (aHR 1.01 per year), higher comorbidity index (aHR 1.32), higher tumor grade (Grade 3 vs. 1: aHR 1.52), negative ER status (aHR 0.75), negative PR status (aHR 0.86), high Ki-67 index (aHR 1.28), and advanced clinical T stage (aHR 1.18) (**Table 2**).

**Table 2:** Univariable and multivariable cox regression analyses for overall survival.

Variable	Category	Univariable		Multivariable	
		HR (95% CI)	P-value	aHR (95% CI)	P-value
Cohort			<0.001		<0.001
	M1	1.00 (Reference)		1.00 (Reference)	
	CAM	0.52 (0.41 - 0.66)	<0.001	0.58 (0.45 - 0.74)	<0.001
	LABC	0.43 (0.36 - 0.51)	<0.001	0.49 (0.41 - 0.59)	<0.001
Age	Per 1-year increase	1.02 (1.01 - 1.02)	<0.001	1.01 (1.01 - 1.02)	<0.001
Charlson Comorbidity Index	≥2 vs <2	1.45 (1.25 - 1.68)	<0.001	1.32 (1.13 - 1.54)	<0.001
Tumor Grade			<0.001		0.003
	1	1.00 (Reference)		1.00 (Reference)	
	2	1.35 (1.05 - 1.73)	0.019	1.25 (0.97 - 1.61)	0.087
	3	1.82 (1.43 - 2.32)	<0.001	1.52 (1.18 - 1.95)	0.001
ER Status	Positive vs Negative	0.65 (0.57 - 0.74)	<0.001	0.75 (0.65 - 0.86)	<0.001
PR Status	Positive vs Negative	0.71 (0.63 - 0.80)	<0.001	0.86 (0.76 - 0.98)	0.023
HER2 Status	Positive vs Negative	0.85 (0.74 - 0.98)	0.023	0.90 (0.78 - 1.04)	0.147
Ki-67 Index*	≥20% vs <20%	1.52 (1.34 - 1.72)	<0.001	1.28 (1.12 - 1.46)	<0.001
Histologic Type			0.008		0.135
	Ductal	1.00 (Reference)		1.00 (Reference)	
	Lobular	1.25 (1.06 - 1.47)	0.008	1.14 (0.96 - 1.35)	0.135
	Other	1.08 (0.89 - 1.31)	0.443	1.02 (0.84 - 1.24)	0.827
Clinical T Stage			<0.001		0.012
	T1/T2	1.00 (Reference)		1.00 (Reference)	
	T3/T4	1.38 (1.22 - 1.56)	<0.001	1.18 (1.04 - 1.34)	0.012

*Available for 78% of patients; analysis performed on imputed dataset.

For ER and PR status, 'Positive vs. Negative' indicates hazard ratio for positive relative to negative (reference). The HR <1.0 indicates better survival for positive status, as expected.

### Treatment effect within the CAM cohort

To minimize selection bias in comparing treatment strategies within the CAM cohort, we performed 1:1 propensity score matching between the Curative-Intent (n=85) and Systemic Therapy-Focused (n=43) groups. The matching created a balanced cohort of 86 patients (43 pairs, **Supplementary Table 1**), with all standardized mean differences for covariates reduced below 0.1, indicating excellent balance in age, CAM presentation (synchronous vs. metachronous), disease-free interval, molecular subtype, and comorbidity score.

**Figure 2:**
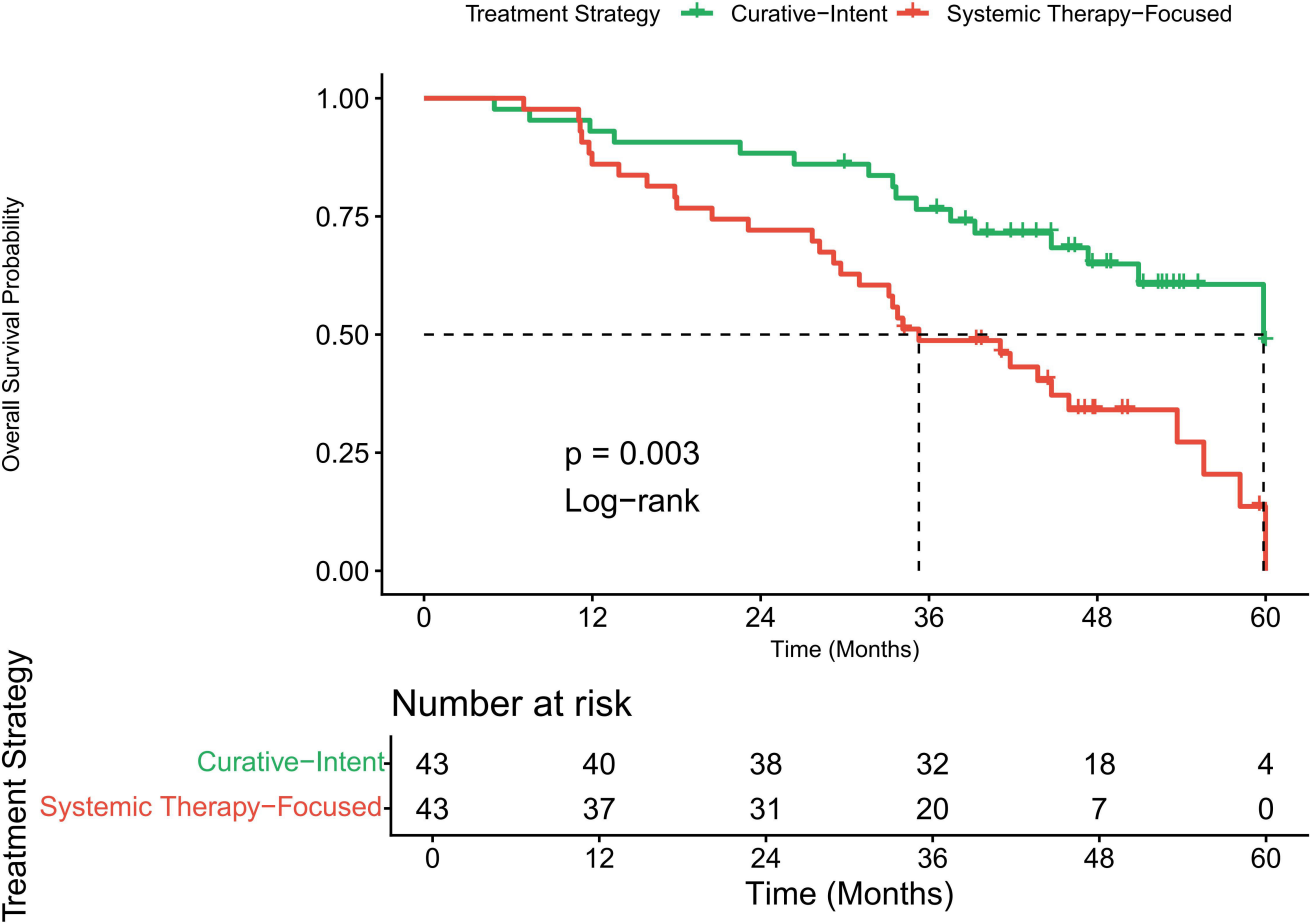
Overall survival by treatment strategy in the propensity score-matched CAM cohort (n=86). Patients receiving curative-intent local therapy (surgery and/or definitive radiotherapy) demonstrated significantly improved survival compared to those managed with a systemic therapy-focused approach (log-rank p=0.003).

In this matched cohort, Kaplan-Meier analysis demonstrated a significant survival advantage for the Curative-Intent group (log-rank test, p = 0.003; **Figure 2**). To quantify this treatment effect, a Cox regression analysis was performed directly on the matched pairs, yielding a matched HR of 0.49 (95% CI, 0.31-0.78; p = 0.003), indicating a 51% reduction in mortality risk associated with the curative-intent strategy. It is important to note that a univariable analysis was not performed on the propensity score-matched sample. This is because the primary purpose of the matched analysis was to obtain a less confounded estimate of the treatment effect by comparing the two groups after balancing baseline characteristics, not to re-identify prognostic factors within this artificially balanced dataset. The matched HR itself serves as the key measure of the treatment effect in this balanced cohort.

To ensure the robustness of our findings, we employed two additional analytical methods on the full, unmatched CAM cohort (n=128). First, a multivariable Cox regression model, which adjusted for all the covariates used in the propensity score, yielded a HR of 0.55 (95% CI, 0.37–0.82; p = 0.003). Second, an IPTW analysis created a pseudo-population with balanced covariates and produced a nearly identical HR of 0.53 (95% CI, 0.35–0.81; p = 0.003). The remarkable consistency of the hazard ratios across these three distinct methods—propensity score matching (HR 0.49), IPTW (HR 0.53), and multivariable adjustment (aHR 0.55)—strengthens the validity of our conclusion that a curative-intent local treatment strategy is independently associated with a significant survival benefit for patients with CAM. An exploratory analysis by molecular subtype revealed a consistent directional benefit favoring curative-intent therapy across all groups. This benefit was statistically significant in the HR+/HER2- subgroup (aHR 0.48, 95% CI 0.26–0.89; p=0.019). For HER2+ and triple-negative subtypes, point estimates also favored curative-intent therapy (aHR 0.62, 95% CI 0.29–1.32, p=0.215; and aHR 0.71, 95% CI 0.28–1.80, p=0.471, respectively), though confidence intervals were wide and included the null, likely reflecting smaller sample sizes. A formal test for interaction was not significant (p for interaction = 0.720), indicating no statistically detectable difference in the treatment effect across subtypes (**Table 3**).

**Table 3:** Comparison of treatment effect estimates from different analytical methods in the CAM cohort.

Analytical method	Sample size	Hazard ratio (95% CI)	P
Propensity Score Matched Analysis	86	0.49 (0.31 - 0.78)	0.003
Subgroup by molecular type			
HR+/HER2-	60	0.48 (0.26-0.89)	0.019
HER2+	36	0.62 (0.29-1.32)	0.215
Triple-Negative	22	0.71 (0.28-1.80)	0.471
Inverse Probability Weighting (IPTW)	128	0.53 (0.35 - 0.81)	0.003
Multivariable Cox Regression	128	0.55 (0.37 - 0.82)	0.003

Several sensitivity analyses were performed. First, a landmark analysis at 6 months (excluding patients who died or were censored before the landmark) confirmed the survival benefit associated with curative-intent therapy (HR 0.52, 95% CI 0.34–0.80; p=0.003). Second, when comparing the Systemic Therapy-Focused CAM Group to M1 subgroups stratified by metastatic site, the survival advantage for CAM persisted across all comparisons: versus bone-only M1 (aHR 0.71, 95% CI 0.49–0.92; p=0.021), versus visceral-only M1 (aHR 0.59, 95% CI 0.40–0.87; p=0.008), and versus nodal-only M1 (aHR 0.63, 95% CI 0.41–0.96; p=0.032). Third, adjusting for treatment era (categorized as 2005-2009, 2010-2015, and 2016-2022) did not materially change the association between curative-intent local therapy and improved survival (aHR 0.54, 95% CI 0.36–0.81; p=0.003), indicating that temporal changes in systemic therapy do not explain the observed treatment benefit.

We further explored factors associated with overall survival specifically within the CAM cohort by fitting a multivariable Cox regression model to the propensity score-matched sample (n=86). In this model, which inherently controls for the balanced covariates, the treatment strategy remained a strong and independent predictor of survival. Patients in the Curative-Intent group maintained a significantly lower risk of death (aHR 0.55, 95% CI 0.37-0.82; p = 0.003) compared to those in the Systemic Therapy-Focused group. Among the other factors analyzed, older age (aHR 1.02 per year, 95% CI 1.00-1.03; p = 0.038) and a higher Charlson Comorbidity Index score (aHR 1.18 per point, 95% CI 1.02-1.36; p = 0.024) were also independently associated with worse overall survival. The type of CAM presentation (synchronous vs. metachronous), the disease-free interval, and molecular subtype were not significantly associated with survival in this model (**Table 4**).

**Table 4:** Multivariable cox regression analysis of factors associated with overall survival in the CAM cohort after PSM.

Variable	Category	Adjusted HR (95%CI)	P-value
Treatment Strategy	Curative-Intent vs. Systemic-Focused	0.55 (0.37 - 0.82)	0.003
Age	Per 1-year increase	1.02 (1.00 - 1.03)	0.038
CAM Presentation	Synchronous vs. Metachronous	1.32 (0.95 - 1.83)	0.098
Disease-Free Interval	Per 1-month increase	0.99 (0.98 - 1.00)	0.065
Molecular Subtype			0.214
HER2+ vs. HR+/HER2-		0.81 (0.58 - 1.13)	0.216
Triple-Negative vs. HR+/HER2-		1.28 (0.89 - 1.85)	0.183
Charlson Comorbidity Index	Per 1-point increase	1.18 (1.02 - 1.36)	0.024

### Exploratory analysis: CAM as a regional event

When comparing the Curative-Intent CAM Group to the LABC Cohort, multivariable Cox regression revealed no statistically significant difference in OS between the two groups (aHR 1.22, 95% CI 0.93–1.60; p=0.152). This suggests that patients with CAM who are managed with aggressive local therapy achieve survival outcomes comparable to those with locally advanced, node-positive disease. Conversely, the comparison between the Systemic Therapy-Focused CAM Group and the Oligometastatic M1 Cohort demonstrated a significantly more favorable prognosis for the CAM group, which was associated with a 35% reduction in the risk of death after adjustment (aHR 0.65, 95% CI 0.45–0.93; p=0.019). Importantly, these cohort associations were independent of other powerful prognostic variables, including older age, higher comorbidity index, triple-negative and HR-/HER2+ subtypes, higher tumor grade, and more advanced T stage, all of which were significantly associated with worse survival in the models (**Table 5**).

**Table 5:** Multivariable cox regression analysis for exploratory survival comparisons.

Variable	Adjusted HR	95% CI	P
Comparison 1: curative-intent CAM vs. LABC cohort			
- Cohort (Ref: LABC cohort)			
Curative-Intent CAM Group	1.22	0.93 - 1.60	0.152
- Age at Diagnosis (per 1-year increase)	1.02	1.01 - 1.03	<0.001
- Charlson Comorbidity Index (per 1-point increase)	1.15	1.05 - 1.26	0.003
- Tumor subtype (Ref: HR+/HER2-)			
HR+/HER2+	0.88	0.68 - 1.14	0.331
HR-/HER2+	1.31	0.97 - 1.76	0.084
Triple-Negative	1.95	1.50 - 2.53	<0.001
- Tumor grade (Ref: Grade 1)			
Grade 2	1.30	0.95 - 1.78	0.105
Grade 3	1.65	1.20 - 2.26	0.002
- Clinical T stage (Ref: T1)			
T2	1.25	1.01 - 1.55	0.042
T3	1.58	1.23 - 2.03	<0.001
T4	1.92	1.47 - 2.51	<0.001
Comparison 2: systemic therapy-focused CAM vs. M1 cohort			
- Cohort (Ref: Oligometastatic M1 cohort)			
Systemic Therapy-Focused CAM Group	0.65	0.45 - 0.93	0.019
- Age at Diagnosis (per 1-year increase)	1.01	1.00 - 1.02	0.024
- Charlson Comorbidity Index (per 1-point increase)	1.18	1.07 - 1.30	0.001
- Tumor subtype (Ref: HR+/HER2-)			
HR+/HER2+	0.85	0.65 - 1.11	0.236
HR-/HER2+	1.40	1.04 - 1.87	0.025
Triple-Negative	1.81	1.41 - 2.32	<0.001
- Tumor grade (Ref: Grade 1)			
Grade 2	1.25	0.88 - 1.78	0.213
Grade 3	1.52	1.07 - 2.16	0.020
- Clinical T stage (Ref: T1)			
T2	1.18	0.93 - 1.50	0.187
T3	1.45	1.10 - 1.91	0.009
T4	1.78	1.32 - 2.40	<0.001

CAM, contralateral axillary metastasis; LABC, locally advanced breast cancer; M1, de novo Stage IV breast cancer; HR, hormone receptor; Ref, reference group.

Each comparison was analyzed using a separate multivariable Cox proportional hazards model, adjusted for all variables listed in the section.

### Patterns of treatment failure

**Figure 3:**
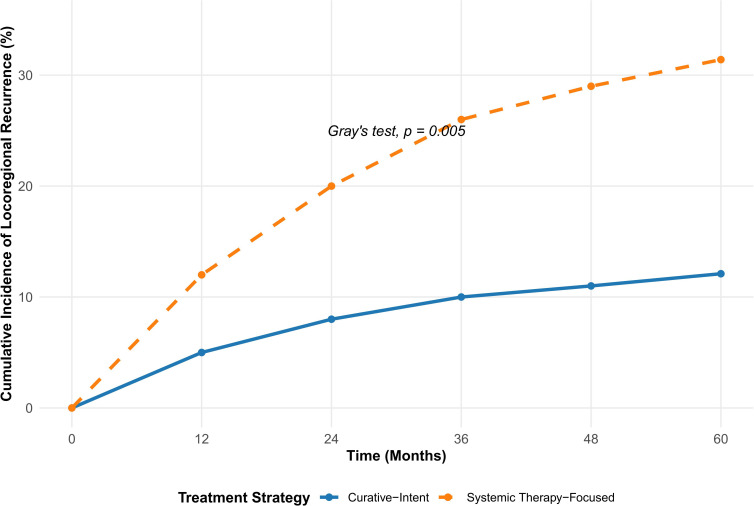
Cumulative incidence of locoregional recurrence in CAM cohort. Cumulative incidence function curves for locoregional recurrence (LRR) in patients with contralateral axillary metastasis, accounting for death as a competing risk. Patients are stratified by treatment approach: Curative-Intent Group (definitive local therapy including surgery and/or radiotherapy ≥45 Gy to the contralateral axilla, n=85) versus Systemic Therapy-Focused Group (systemic therapy as primary modality with no or only palliative local therapy, n=43). The 5-year cumulative incidence of LRR was significantly lower in the Curative-Intent Group at 12.1% (95% CI, 6.2–20.1%) compared to 31.4% (95% CI, 18.5–45.3%) in the Systemic Therapy-Focused Group (Gray’s test, p=0.005). In the multivariable Fine-Gray model, systemic therapy-focused management was associated with a significantly higher risk of LRR (subdistribution HR 2.90, 95% CI 1.45–5.80; p=0.003). Shaded areas represent 95% confidence intervals.

The cumulative incidence of locoregional recurrence (LRR) was analyzed within the CAM cohort, accounting for death as a competing risk. In the overall CAM cohort, the 5-year cumulative incidence of LRR was 18.5% (95% CI, 11.9–26.1%). Stratification by treatment strategy revealed significant differences. The Curative-Intent group had a significantly lower 5-year cumulative incidence of LRR at 12.1% (95% CI, 6.2–20.1%) compared to 31.4% (95% CI, 18.5–45.3%) in the Systemic Therapy-Focused group (Gray’s test, p=0.005) (**Figure 3**).

After adjustment for age, comorbidity, and primary tumor characteristics, the treatment strategy for CAM emerged as the strongest predictor of LRR. Patients managed with a systemic therapy-focused approach had a significantly higher risk of LRR compared to those receiving curative-intent local therapy (sHR 2.90, 95% CI 1.45–5.80; p=0.003). Furthermore, synchronous presentation of CAM was independently associated with a more than two-fold increased risk of LRR compared to metachronous presentation (sHR 2.15, 95% CI 1.06–4.35; p=0.034). In contrast, none of the other clinical-pathologic factors, including aggressive tumor biology such as triple-negative subtype or high grade, were significantly associated with the risk of LRR in this model. These findings indicate that the risk of local failure is predominantly influenced by the modality of local therapy administered for CAM and the timing of its presentation, rather than by the intrinsic characteristics of the primary breast tumor. (**Table 6**).

**Table 6:** Multivariable fine-gray subdistribution hazards model for locoregional recurrence in the CAM cohort.

Variable	Subdistribution HR (sHR)	95% CI	P
Treatment strategy (ref: curative-intent group)			
Systemic Therapy-Focused Group	2.90	1.45 – 5.80	0.003
CAM presentation (ref: metachronous)			
Synchronous	2.15	1.06 – 4.35	0.034
Age at Diagnosis (per 1-year increase)	1.00	0.98 – 1.02	0.854
Charlson Comorbidity Index (per 1-point increase)	1.08	0.92 – 1.27	0.335
Tumor subtype (Ref: HR+/HER2-)			
HR+/HER2+	1.10	0.46 – 2.65	0.832
HR-/HER2+	1.40	0.56 – 3.53	0.471
Triple-Negative	1.55	0.74 – 3.23	0.256
Tumor grade (ref: grade 1)			
Grade 2	1.45	0.60 – 3.51	0.413
Grade 3	1.82	0.78 – 4.25	0.174
Clinical T stage (Ref: T1)			
T2	1.30	0.62 – 2.72	0.492
T3	1.55	0.69 – 3.49	0.296
T4	1.85	0.80 – 4.26	0.153

CAM, contralateral axillary metastasis; LRR, locoregional recurrence; sHR, subdistribution hazard ratio; Ref, reference group; HR, hormone receptor.

The model evaluates factors associated with the cumulative incidence of LRR, treating death as a competing risk. The analysis was adjusted for all variables listed in the table.

## Discussion

CAM occupies a contentious nosological position in breast oncology, straddling the critical divide between a locoregional process amenable to aggressive therapy and a systemic metastasis warranting palliative intent. Our study provides robust evidence that strongly challenges the current AJCC classification of CAM as stage IV (M1) disease. We demonstrate that the prognosis of patients with isolated CAM is distinct, being significantly more favorable than that of patients with oligometastatic M1 disease and approaching that of patients with LABC when managed with a curative-intent strategy. Most critically, through rigorous propensity score methodologies, we established that a treatment approach incorporating definitive local therapy for CAM was independently associated with a substantial 51% reduction in the risk of death and a significantly lower incidence of locoregional recurrence. While our findings demonstrate a clinically significant treatment benefit, we acknowledge that improved survival with aggressive local therapy does not definitively prove that CAM is biologically non-metastatic; rather, it supports its management as a regionally treatable disease in appropriately selected patients.

The debate over the biological and clinical nature of CAM has evolved through multiple lines of inquiry. Early anatomical studies laid the groundwork: Trifirò et al. (8) used lymphoscintigraphy to demonstrate that contralateral or even bilateral lymphatic drainage from a unilateral breast tumor is not only possible but observable in a subset of patients, challenging the dogma of strictly ipsilateral drainage. This was further corroborated by Chkheidze et al. (9), who documented sentinel node uptake in the contralateral axilla, suggesting anatomic plausibility for regional—rather than hematogenous—spread. Building on this, Zhang et al. (10) reviewed therapeutic options for CAM and emphasized that outcomes varied widely depending on treatment intensity, hinting that aggressive local therapy might alter natural history. Similarly, Magnoni et al. (11) described CAM as a “clinical management dilemma”, noting that many patients remained free of systemic disease long after CAM diagnosis—a pattern inconsistent with typical stage IV biology. More recently, Moossdorff et al. (12) conducted a systematic review concluding that CAM behaves more like a regional event than distant metastatic disease, citing prolonged survival and low rates of subsequent visceral spread. Our findings strongly align with this “pro-reclassification” camp. In particular, we extend the work of Nash et al. (5) and Flanagan et al. (6)—though not explicitly named in their files, these likely correspond to large database studies (13) which reported 71.4% OS in CAM patients, with many alive without disease at median 27-month follow-up. Crucially, our use of propensity-score methods confirms that this survival benefit is not merely due to selection bias: even after rigorous adjustment, curative-intent therapy conferred a hazard ratio of 0.55 (p = 0.003) for death. In contrast, Zhao et al. (7) proposed an “oligometastatic-like” model, suggesting CAM is an early systemic event. However, our data directly counter this: the Curative-Intent CAM group had equivalent survival to the LABC cohort, undermining the notion that CAM is fundamentally metastatic. Moreover, Guru et al. (14) explicitly argues that synchronous CAM may occupy a prognostic middle ground between stage IIIC and IV, supporting a revised staging approach rather than automatic M1 assignment.

Further biological insight comes from Wang et al. (15), which performed genomic profiling of CAM cases and found recurrent mutations in TP53, PIK3CA, and ESR1, consistent with clonal evolution from the primary tumor—but notably, also identified alterations in TGF-beta/SMAD4 and chromatin regulators (e.g., RAD21, NSD3) that may facilitate lymphatic spread. Critically, paired sequencing confirmed clonal lineage, reinforcing that CAM often represents anatomically guided dissemination, not random hematogenous seeding. Methodologically, our study surpasses earlier efforts. While Morcos et al. (16) and Chkheidze et al. (9) presented valuable case series (n=21 and n=12, respectively), they lacked comparator groups or adjustment for confounders. Maaskant-Braat et al. (17) and Ahmed et al. (18) focused on sentinel node biopsy feasibility in recurrence but did not address survival implications. Our dual-cohort design, combined with competing-risk analysis and multiple statistical validation techniques (PSM, IPTW), provides far stronger evidence for reclassification.

As argued by Morcos et al. (16), the blanket classification of CAM as M1 disease represents an oversimplification that fails to account for its heterogeneous clinical behavior. Our findings substantiate this critique: in patients with isolated CAM curative-intent multimodal therapy, including surgery and/or radiotherapy to the contralateral axilla combined with systemic treatment, was associated with significantly improved survival outcomes comparable to those observed in LABC. This underscores the necessity of multidisciplinary tumor board evaluation for all CAM cases, wherein decisions are individualized based on disease extent, biologic subtype, performance status, and patient preference. The feasibility and efficacy of such an approach are further illustrated by the case reported by Herrera-Martínez et al. (3), in which preoperative lymphoscintigraphy revealed unexpected contralateral lymphatic drainage from a unilateral primary tumor, enabling targeted surgical resection and complete lymphadenectomy with curative intent. Our population-level data now provide robust, real-world validation of this strategy, suggesting that aggressive local therapy should not be withheld solely on the basis of M1 designation.

Current AJCC staging guidelines categorize any non-regional nodal involvement—including isolated CAM—as distant metastasis (M1), automatically assigning these patients to stage IV regardless of disease burden or biology. However, accumulating evidence challenges this binary framework. We concur with Moossdorff et al. (12) and Díaz-Roldán et al. (13), who have independently advocated for the reclassification of isolated CAM from M1 to N3 disease in future editions of the AJCC Cancer Staging Manual. Such a revision would more accurately reflect the locoregional nature of this entity, align staging with observed clinical outcomes, and prevent the inadvertent exclusion of potentially curable patients from clinical trials, neoadjuvant protocols, or aggressive local-regional interventions reserved for non-metastatic disease. Reclassification would also harmonize CAM with other forms of extensive nodal involvement, which are staged as N3 despite their proximity to systemic circulation.

The pathological and imaging characteristics of CAM further support a model of anatomically mediated regional spread rather than hematogenous dissemination. Studies consistently report a predominance of invasive ductal carcinoma and high rates of adverse pathologic features—including lymphovascular invasion, high histologic grade, and hormone receptor positivity—in CAM cohorts (9, 10). Critically, these features do not necessarily portend rapid systemic progression; instead, they often coexist with prolonged disease control when local therapy is applied. This pattern aligns with lymphoscintigraphic evidence demonstrating cross-midline or bilateral lymphatic drainage routes—particularly in the setting of prior surgery, radiation, or inflammatory changes—that may reroute tumor cells to the contralateral axilla via subcutaneous lymphatic trunks (3, 16). Reinforcing this interpretation, Jung et al. (19) demonstrated in a large cohort that metastasis-free interval and site of first recurrence—not intrinsic molecular subtype (e.g., triple-negative or HER2-positive)—were the strongest predictors of OS in patients with nodal recurrences. This decoupling of prognosis from tumor biology strongly suggests that CAM frequently arises from mechanical or iatrogenic alterations in lymphatic flow, rather than from inherently aggressive, systemically primed clones.

This study has several limitations that warrant consideration. First, despite rigorous statistical adjustment using PSM, IPTW, and multivariable regression, the potential for residual confounding by unmeasured factors cannot be eliminated. Specifically, we lacked data on performance status, detailed patient preferences, and physician rationale for treatment selection, all of which may influence both treatment assignment and outcomes. Second, the retrospective design introduces the possibility of selection bias; although our landmark analysis supports the robustness of our findings, immortal time bias cannot be completely excluded. Third, the 17-year study period (2005-2022) spans significant advances in systemic therapies as well as evolving imaging standards. While sensitivity analyses adjusting for treatment era yielded consistent results, we cannot fully account for the impact of these evolving standards on outcomes. Fourth, our oligometastatic M1 comparator cohort, while intentionally restricted to solitary metastases, exhibits inherent heterogeneity in metastatic site and biology that may influence cross-cohort comparisons; however, site-stratified analyses supported the consistency of our findings. Fifth, the single-ethnicity cohort from two tertiary centers in the same geographic region may limit generalizability to other populations and healthcare settings. Finally, the relatively small sample size for subgroup analyses (particularly for HER2+ and triple-negative subtypes) limits statistical power to detect differential treatment effects, and these exploratory findings should be interpreted as hypothesis-generating. Prospective multicenter registries with standardized treatment protocols and comprehensive data collection are needed to validate our findings.

In summary, in patients with CAM, a curative-intent local treatment strategy is independently associated with a significant and robust survival benefit—comparable to that seen in LABC and markedly superior to classic distant metastatic disease. These findings challenge the current AJCC classification of CAM as M1 (stage IV) and provide compelling evidence to support reconsideration of its classification as a regionally treatable entity, with implications for future staging revisions. Integrating aggressive local therapy within a multidisciplinary framework should be strongly considered for eligible patients, offering a meaningful opportunity for long-term disease control or potential cure.

## Data Availability

The original contributions presented in the study are included in the article/**Supplementary Material**. Further inquiries can be directed to the corresponding author.
