# Supplementary material for: Reclassifying contralateral axillary metastasis: survival benefit of aggressive local therapy challenges stage IV designation
Source: Front Oncol. 2026 Apr 16;16:1748851. doi: 10.3389/fonc.2026.1748851 (PMC13128415; doi:10.3389/fonc.2026.1748851)
Supplement: Supplementary file 1 [file SupplementaryFile1.doc]

****Supplementary Table 1: Baseline Characteristics of the Propensity-Score Matched CAM Cohort (n=86)****

| **Characteristic** | **Curative-Intent Group (n=43)** | **Systemic Therapy-Focused Group (n=43)** | **P-value** | **SMD** |
| --- | --- | --- | --- | --- |
| ****Molecular Subtype, n (%)**** |  |  | 0.842 | 0.08 |
| HR+/HER2- | 21 (48.8%) | 19 (44.2%) |  |  |
| HER2+ | 11 (25.6%) | 13 (30.2%) |  |  |
| Triple-Negative | 8 (18.6%) | 7 (16.3%) |  |  |
| Other/Unknown | 3 (7.0%) | 4 (9.3%) |  |  |
| ****Age, median (IQR)**** | 56 (48, 64) | 55 (49, 63) | 0.912 | 0.02 |
| ****Synchronous CAM, n (%)**** | 16 (37.2%) | 15 (34.9%) | 1.000 | 0.05 |
| ****DFI (mo), median (IQR)**** | 40 (26, 65) | 43 (30, 70) | 0.567 | 0.10 |
| ****Charlson Index ≥2, n (%)**** | 5 (11.6%) | 6 (14.0%) | 1.000 | 0.07 |

Abbreviations: PSM, propensity score matching; SMD, standardized mean difference; HR, hormone receptor; CAM, contralateral axillary metastasis; DFI, disease-free interval.
Note: This table confirms excellent balance in molecular subtypes and other key covariates after matching.
